# Association between the oxytocin receptor (*OXTR*) gene and mesolimbic responses to rewards

**DOI:** 10.1186/2040-2392-5-7

**Published:** 2014-01-31

**Authors:** Cara R Damiano, Joseph Aloi, Kaitlyn Dunlap, Caley J Burrus, Maya G Mosner, Rachel V Kozink, Ralph Edward McLaurin, O’Dhaniel A Mullette-Gillman, Ronald McKell Carter, Scott A Huettel, Francis Joseph McClernon, Allison Ashley-Koch, Gabriel S Dichter

**Affiliations:** 1 Department of Psychology, University of North Carolina, CB#3270, Davie Hall, UNC-CH, Chapel Hill, NC 27599, USA; 2 Brain Imaging and Analysis Center (BIAC), Duke University, Durham, NC, USA; 3 Department of Psychiatry and Behavioral Sciences, Duke University, Durham, NC, USA; 4 Department of Neurobiology, Duke University, Durham, NC, USA; 5 Neuroscience and Behavioral Disorders Program, Duke-NUS Graduate Medical School, Singapore; 6 Psychology Department, National University of Singapore, Singapore; 7 Center for Cognitive Neuroscience, Duke University, Durham, NC, USA; 8 Departments of Psychology and Neuroscience, Duke University, Durham, NC, USA; 9 Department of Medicine, Center for Human Genetics, Duke University, Durham, NC, USA; 10 Department of Psychiatry, University of North Carolina, Chapel Hill, NC, USA; 11 Carolina Institute for Developmental Disabilities, University of North Carolina, Chapel Hill, NC, USA

**Keywords:** Autism spectrum disorder (ASD), Oxytocin, Oxytocin receptor, Genetics, Neuroimaging, Reward, Motivation, Mesolimbic, Functional magnetic resonance imaging (fMRI), Single nucleotide polymorphism (SNP)

## Abstract

**Background:**

There has been significant progress in identifying genes that confer risk for autism spectrum disorders (ASDs). However, the heterogeneity of symptom presentation in ASDs impedes the detection of ASD risk genes. One approach to understanding genetic influences on ASD symptom expression is to evaluate relations between variants of ASD candidate genes and neural endophenotypes in unaffected samples. Allelic variations in the oxytocin receptor (OXTR) gene confer small but significant risk for ASDs for which the underlying mechanisms may involve associations between variability in oxytocin signaling pathways and neural response to rewards. The purpose of this preliminary study was to investigate the influence of allelic variability in the OXTR gene on neural responses to monetary rewards in healthy adults using functional magnetic resonance imaging (fMRI).

**Methods:**

The moderating effects of three single nucleotide polymorphisms (SNPs) (rs1042778, rs2268493 and rs237887) of the OXTR gene on mesolimbic responses to rewards were evaluated using a monetary incentive delay fMRI task.

**Results:**

T homozygotes of the rs2268493 SNP demonstrated relatively decreased activation in mesolimbic reward circuitry (including the nucleus accumbens, amygdala, insula, thalamus and prefrontal cortical regions) during the anticipation of rewards but not during the outcome phase of the task. Allelic variation of the rs1042778 and rs237887 SNPs did not moderate mesolimbic activation during either reward anticipation or outcomes.

**Conclusions:**

This preliminary study suggests that the OXTR SNP rs2268493, which has been previously identified as an ASD risk gene, moderates mesolimbic responses during reward anticipation. Given previous findings of decreased mesolimbic activation during reward anticipation in ASD, the present results suggest that OXTR may confer ASD risk via influences on the neural systems that support reward anticipation.

## Background

Autism spectrum disorders (ASDs) are highly heritable neurodevelopmental disorders [1] and there has been significant progress in identifying genes that confer increased risk for ASDs [2,3]. However, finding clear associations between genetic risk variants and ASD symptom expression has been challenging due to the highly heterogeneous nature of the disorder. One approach to address these challenges and improve our understanding of genetic influences on ASD symptom expression is to evaluate relations between variants of ASD candidate genes and ASD-relevant neural endophenotypes in homogeneous unaffected samples [4]. This imaging-genetics approach has the potential to elucidate genetic influences on neural endophenotypes relevant to the expression of ASD symptoms [5].

Although there are hundreds of identified ASD risk genes [6-8], the oxytocin receptor (OXTR) gene has been shown to confer a small but significant risk for ASDs. The influence of the OXTR gene in ASDs has been established across different ethnic populations and different study methodologies (genome-wide association studies and linkage analyses) [9-12]. There is also evidence of altered methylation of the OXTR promoter gene and reduced expression of OXTR in the post-mortem brains of individuals with ASDs, providing further evidence for the role of OXTR, the oxytocin (OXT) signaling pathway and epigenetic regulation of OXTR in the etiology of ASDs [13].

OXT is a neuropeptide that has been consistently linked to affiliative and social behaviors in preclinical and human studies [14-17]. There is accumulating evidence for the role of altered OXT functioning in the etiology of social impairments in ASDs [18]: atypical levels of plasma OXT and its precursors have been found in children with ASDs [19,20] and exogenous OXT administration has been shown to improve social functioning in children and adults with ASDs [21-23].

However, the potential mechanisms by which OXT influences social functioning in ASDs remain poorly understood. One possibility is via effects of the OXT signaling pathways on mesolimbic brain systems that code for the motivational relevance of social rewards [24,25]. There has recently been increased interest in examining the effect of motivational factors on social functioning in ASDs. The social motivation hypothesis of ASD posits that disruption of neural mechanisms supporting social motivation may constitute a primary deficit in ASDs with potential downstream effects on the development of social cognition [26,27]. This model is supported by initial neuroimaging evidence for attenuated neural responses to social rewards in mesolimbic brain regions in ASDs [28,29], though there is evidence of altered mesolimbic functioning in response to non-social rewards in ASDs as well [30,31].

Brain regions that code for reward valuation, including the nucleus accumbens and amygdala, have dense OXTR expression [32,33] and OXT binding in these regions influences social behaviors [34-39]. Additionally, OXT plays a key role in regulating dopaminergic activity in mesolimbic reward circuitry [40-43]. Infusion of an OXT agonist into the ventral tegmental area results in increased dopaminergic neuron firing in the ventral striatum in a manner that predicts variability in maternal behavior [41], and administration of intranasal OXT enhances mesolimbic activation in response to social rewards (for example, viewing a smiling infant face) [44,45]. Additionally, plasma levels of OXT are associated with variability in ventral and dorsal striatal activation during reciprocated cooperation, a socially rewarding experience [45,46]. Variants of the OXTR gene are associated with structure and function of reward-related brain regions, including the hypothalamus, insula and amygdala [47-49]. These studies suggest that OXT may stimulate mesolimbic dopamine release, which may thereby facilitate approach behaviors towards rewards [50].

Despite the central role of OXT systems for social motivation and evidence for OXT binding in mesolimbic reward-processing brain regions, no study to date has examined the effects of individual differences in OXT functioning on mesolimbic responses to rewards. The present study examined how allelic variants of the OXTR gene influence neural responses to rewards during functional magnetic resonance imaging (fMRI). To index the neural substrates of reward processing, we used a monetary incentive delay (MID) task [51] that reliably recruits reward-processing circuitry across a wide range of clinical and non-clinical populations [52-59]. This preliminary investigation addressed only responses to monetary rewards since we were primarily interested in testing the hypothesis that OXTR variants are generally associated with dopaminergic mesolimbic activation and monetary rewards are a common laboratory proxy for primary rewards that are known to activate mesolimbic brain regions robustly [60].

Three OXTR single nucleotide polymorphisms (SNPs) were examined (rs1042778, rs2268493 and rs237887), each of which has previously been linked to individual differences in social functioning and ASD risk. The rs1042778 SNP has been associated with ASD risk as well as the severity of social impairments in ASDs [10], prosocial decision-making in typical controls [61], and social-communicative abilities in individuals with ASDs and their families [62]. The rs2268493 SNP has been linked to increased risk for ASDs and increased social impairment, communicative symptoms and restricted and repetitive behaviors in ASDs [9,10]. Finally, the rs237887 SNP has been associated with prosocial decision-making [61] and empathy [63] in typical controls and has been associated with an increased risk of ASDs in the Japanese population [64]. Our overarching hypothesis was that OXTR alleles previously linked to ASD risk or impaired social functioning would be associated with attenuated mesolimbic activation during reward anticipation specifically. This hypothesis is based on the regulatory effects of OXT specifically on striatal dopamine [40-43], which is a central neurotransmitter that codes for the motivational salience of anticipated rewards [65].

## Methods

### Participants

Initially, 41 healthy adults who had participated in a prior imaging-genetics study at Duke University were contacted to participate. Of these, 36 participants completed the fMRI scan, and five of these were excluded due to unusable genetic information, resulting in a final sample of 31 participants (14 male, see Table 1 for demographic information). Participants were right-handed, were not taking any psychotropic medications and had no current or past psychiatric disorders according to the Structured Clinical Interview for DSM-IV Axis I Disorders (SCID-I) [66], which was administered by a trained interviewer and supervised by a licensed psychologist. All participants scored below the clinical cut-off for the Symptom Checklist-90-R (SCL-90-R) [67,68], a general measure of clinical distress or psychopathology. Participants also completed the National Adult Reading Test – Revised (NART-R). Intelligence quotient (IQ) [69] and the autism spectrum quotient [70], a continuous measure of autism symptomatology, were estimated. To minimize effects of population stratification, the sample included only non-Hispanic Caucasian participants. This study was reviewed and approved by the Duke University Institutional Review Board and written informed consent was obtained from all participants.

**Table 1:** Participant characteristics

**Characteristic**	**Mean**	**Standard deviation**	**Range**
Age	23.58	3.15	19 to 31
Full scale IQ^a^	109.41	2.46	103.75 to 112.40
Verbal IQ^a^	107.98	2.88	100.96 to 112.68
Performance IQ^a^	109.62	1.36	106.31 to 111.84
SCL-90-R GSI^b^	0.18	0.14	0.02 to 0.47
AQ^c^	13.03	5.85	2 to 26
Sex	17 female, 14 male		

^a^Estimated from the National Adult Reading Test – Revised (NART-R).

^b^GSI, Global Severity Index; IQ, intelligence quotient; SCL-90-R, Symptom Checklist-90-Revised (clinical cut-off = 2.49 [68]). ^c^AQ, autism spectrum quotient.

### Genotyping

The Oragene DISCOVER (OGR-250) collection kit (formerly known as the Oragene DNA collection kit) (DNA Genetic Inc, Kanata, ON, Canada) was used to obtain genetic information. All participants provided at least 5 mL of saliva for the genetic sample. DNA was isolated using QIAGEN Autopure LS (Venlo, The Netherlands). As part of a larger study investigating a custom set of SNPs, genotyping was performed by the Center for Human Genome Variation at Duke University using a GoldenGate assay with the Illumina BeadXpress platform (Illumina, Inc, San Diego, CA, USA).

This procedure provided allelic information for the following OXTR SNPs: rs53576, rs237885, rs237887, rs237900, rs1042778, rs2254295, rs2268493, rs4686301, rs9840864 and rs11131149. The available OXTR variants with intact SNP data (call rates >0.9) for all 31 participants included four SNPs: rs237887, rs1042778, rs2254295 and rs2268493. From these four SNPs, three SNPs (rs1042778, rs2268493 and rs237887) were selected based on minor allele frequencies >0.20 in the present sample (the minor allele frequency for the rs2254295 SNP was 0.12). These three SNPs have also been associated with individual differences in social functioning and/or ASD risk in previous studies. For example, see [9,10,62]. Allelic frequencies in these SNPs were similar to the minor allele frequency expected in Caucasian samples and were consistent with Hardy–Weinberg equilibrium (rs2268493: *χ*^2^ = 0.03; rs1042778: *χ*^2^ = 0.03; rs237887: *χ*^2^ = 0.05, in the present sample).

Across all SNPs, risk allele homozygotes were compared to a combined group of non-risk allele homozygotes and heterozygotes. For rs2268493, the T allele has been associated with ASD risk [9,10], and thus we compared 14 risk allele homozygotes (TT) to a combined group of 17 non-risk allele homozygotes and heterozygotes (CC/CT). For rs237887, the A allele has been linked to increased risk for ASD in the Japanese population [64] as well as decreased prosociality [61], and thus we compared nine risk allele homozygotes (AA) to a combined group of 22 heterozygotes and non-risk allele homozygotes (AG/GG). For rs1042778 the G allele has been linked to ASD risk [10,62], and thus we compared 14 risk allele homozygotes (GG) to 17 non-risk allele homozygotes and heterozygotes (TT/TG).

Participants included in the current study were drawn from a larger sample, which included 508 Caucasian participants. To evaluate potential linkage disequilibrium among the three OXT SNPs examined in the present study (rs1042778, rs2268493 and rs237887), we calculated linkage disequilibrium in the larger sample from which these participants were drawn. These SNPs were not in linkage disequilibrium in this larger sample, since in all cases *r*^2^ < 0.64:

•rs1042778 and rs2268493: *r*^2^ = 0.078

•rs1042778 and rs237887: *r*^2^ = 0.291

•rs2268493 and rs237887: *r*^2^ = 0.211

•rs2268493 and rs1042778: *r*^2^ = 0.078

•rs237887 and rs1042778: *r*^2^ = 0.291

•rs237887 and rs2268493: *r*^2^ = 0.211

These were consistent with linkage disequilibrium values from the HapMap website [71].

### fMRI task

Participants completed two runs of a monetary incentive delay (MID) task [57] as part of a 1-hr-long fMRI scan session. During this scan session, the participants also completed two runs of an unrewarded go/no-go task. The MID task and the go/no-go task were presented in a pseudo-randomized, counterbalanced order, and only the results of the MID task are presented here.

Each MID trial consisted of: (1) a 2,000-ms cue indicating whether a monetary reward could be won (a triangle) or not (a circle) on a given trial; (2) a 2,000 to 2,500 ms crosshair fixation; (3) a target bullseye presented for up to 500 ms that required a speeded button press; (4) 3,000 ms of feedback to indicate if the participant gave a sufficiently fast response and (5) a variable length inter-trial interval crosshair. The total duration of the trial was 12 s. For trials in which a monetary reward was possible (incentive trials), a sufficiently fast response resulted in the presentation of an image representing a gain of $1 per successful trial, whereas a slower response resulted in presentation of an ‘X’ indicating that no money had been won. For trials in which a monetary reward was not possible (non-incentive trials), the participants were still instructed to respond as quickly as possible to the bullseye image. During these non-incentive trials, a sufficiently fast response resulted in the presentation of a checkmark symbol indicating a successful response and no monetary gain, whereas a slower response resulted in the presentation of an ‘X’ indicating an unsuccessful response and no monetary gain. Potential reward and non-reward trials were aperiodic and pseudo-randomly ordered. Each run included 40 trials (50% reward trials, 50% non-reward trials). The participants were instructed to try to be successful on as many trials as possible, and that success was contingent on response times. The response time threshold for a reward trial was adaptive to individual differences in response times so that all participants were successful for approximately two-thirds of trials (that is, approximately 66.67% accuracy; actual average accuracy was 66.85%, SD = 0.04). All stimuli were presented using E-Prime presentation software (Psychology Software Tools Inc, Pittsburgh, PA, USA) and were viewed through magnet-compatible goggles (Resonance Technology Inc, Northridge, CA, USA).

### fMRI collection and analysis

Scanning was performed with a General Electric Health Technologies 3-T MR750 scanner (General Electric, Waukesha, WI, USA) with 50-mT.m^-1^ gradients at a 200 T.m^-1^.s^-1^ slew rate. Foam cushions were placed around the head to minimize motion. A 32-channel head coil was used for parallel imaging with a sparse matrix in *k*-space [72] to correct for geometric distortions without the need for an additional field mapping sequence. High-resolution structural images with 162 slices were acquired using a three-dimensional fast SPGR BRAVO pulse sequence (repetition time: 7.584 ms; echo time: 2.936 ms; field of view: 256 mm; image matrix: 256 × 256; voxel size: 1 mm^3^; flip angle: 12º) and used for co-registration with the functional data. Structural images were aligned in the near-axial plane defined by the anterior and posterior commissures. Whole-brain functional images consisted of 32 interleaved slices parallel to the anterior and posterior commissure plane using a SENSE spiral pulse sequence sensitive to blood oxygenation level dependent (BOLD) contrast (repetition time: 2000 ms; echo time: 30 ms; field of view: 24 cm; image matrix: 64 × 64; voxel size: 4 mm^3^; flip angle: 77º). All runs began with four discarded radio-frequency excitations to achieve steady-state equilibrium. No participant had greater than 3 mm movement in the *x*, *y* or *z* dimension.

Functional data were preprocessed using FEAT (FMRI Expert Analysis Tool) [73,74] from FMRIB's software library (FSL) (http://www.fmrib.ox.ac.uk/fsl). Preprocessing for all functional data involved the following steps: (1) brain extraction to remove all non-brain data [75]; (2) motion correction using MCFLIRT [76]; (3) spatial smoothing using a Gaussian kernel of full width half maximum 5 mm; (4) mean-based intensity normalization of all volumes by the same factor and (5) high-pass temporal filtering to remove slow signal drift (60 s) [76]. Functional and structural images were co-registered in native space and normalized to a standard stereotaxic space (using Montreal Neurological Institute templates). All registrations were carried out using the FMRIB Linear Image Registration Tool (FLIRT) for linear registration (affine with 12 degrees of freedom). The same transformation matrices used for structural-to-standard transformations were then used for functional-to-standard space transformations of the co-registered functional images. Registrations were applied using an intermodal registration tool [74,76] and voxel-wise temporal autocorrelation was estimated and corrected using FMRIB's Improved Linear Model [77].

Initial analyses examined task effects in the entire group to verify the activation of the hypothesized mesolimbic reward circuitry by the task. Next, the primary research questions were addressed by examining the moderating effects of allelic group on task-specific brain activation. Data from anticipation and outcome phases were analyzed separately. During the anticipation phase (the time between presentation of cues and potential reward receipt), the contrast of interest was the effect of allelic group on differences in brain activation for incentive versus non-incentive trials. During the outcome phase (during presentation of feedback), the contrast of interest was the effect of allelic groups on differences in brain activation for reward versus non-reward trials. Models were evaluated separately for each of the three SNPs examined.

A double-γ function modeled the hemodynamic response for each event type (incentive/non-incentive trials and reward/non-reward trials) in each run. Using a fixed-effects general linear model, parameter estimates from the two runs from each participant were combined. These data were then used in a random-effects analysis to compare allelic groups, using FMRIB Local Analysis of Mixed Effects (FLAME) [75,78]. Initial whole-brain analyses examining task effects in the entire sample used *Z* statistic images cluster thresholded at *Z* > 5.25 with a corrected cluster significance threshold of *P* < 0.05. This somewhat high *Z* threshold was used to allow visualization of effects primarily in reward-processing regions. For the analyses involving the three individual SNPs, whole-brain analyses were conducted using *Z* statistic images thresholded at *Z* > 3.0 with a corrected cluster significance threshold of *P* < .05 / 3 = 0.0167. Activation localizations were based on Harvard–Oxford cortical and subcortical structural probabilistic atlases, as implemented in FSLView v3.1.8.

## Results

### Genotypic and sex effects on demographic and behavioral data

**Figure 1:**
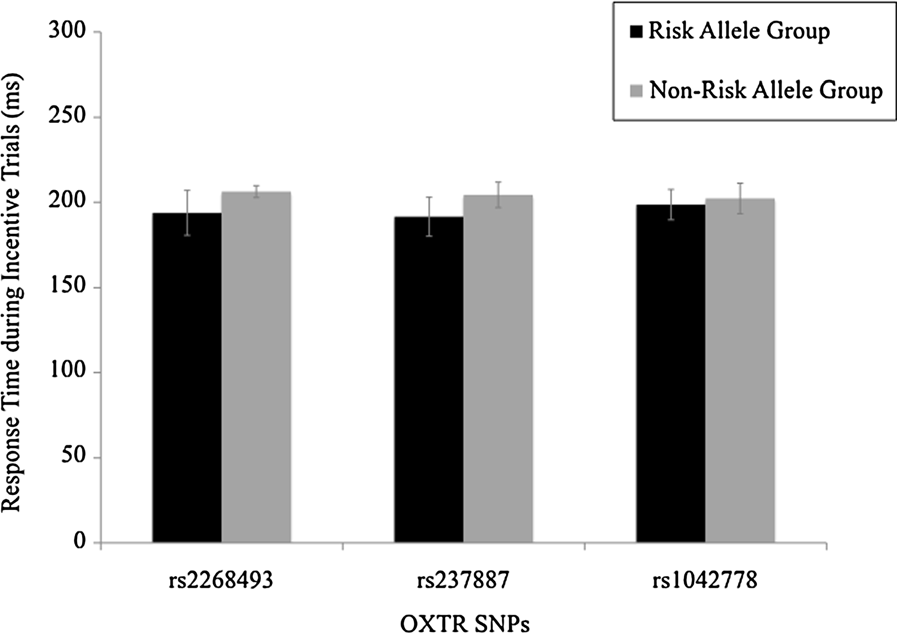
**Response times (in ms) during incentive trials of the monetary incentive delay (MID) task for different oxytocin receptor (OXTR) gene single nucleotide polymorphisms (SNPs) grouped based on rs2268493, rs237887 and rs1042778 allelic variants.** OXTR, oxytocin receptor; SNP, single nucleotide polymorphism.

Potential demographic differences between allelic groups were examined for each SNP. Across all three SNPs, there were no group differences in age, sex ratios, estimated IQ (performance, verbal or full scale) or general clinical symptomatology (based on SCL-90 scores), *P* > .16. There were also no significant differences between allelic groups in response times during the scanner task, *P* > .20 (see Figure 1). In addition, there were also no significant sex differences on any of these demographic variables, *P* > .06, and there were no significant sex differences in response times, *P* > .83. The mean response time across all participants was 212.70 ms (SD = 31.37).

### fMRI data: anticipation phase

#### Entire sample

**Figure 2:**
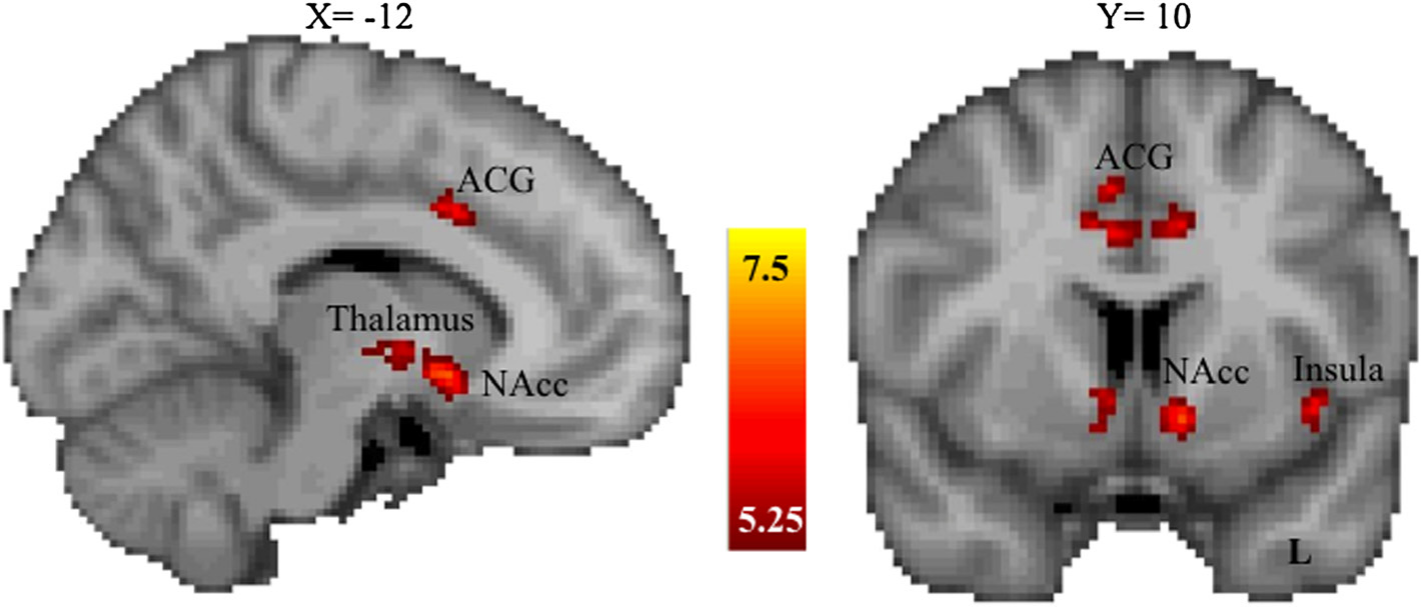
**Brain regions showing significant activation for incentive versus non-incentive trials during the anticipation phase of the monetary incentive delay task across the entire sample.***Z* values indicate cluster thresholds. ACG, anterior cingulate gyrus; L, left; NAcc, nucleus accumbens.

Across the entire sample, regardless of allelic group, reward anticipation activated the bilateral nucleus accumbens as well as other reward-processing regions, including the right caudate, right amygdala, left insular cortex, right thalamus, right anterior cingulate gyrus and left orbital frontal cortex (see Table 2 and Figure 2). Table 2 shows activation during incentive versus non-incentive trials of the anticipation phase of the MID task across the entire sample (thresholded at *Z* > 5.25, corrected cluster significance threshold of *P* < .05).

**Table 2:** Activation during incentive versus non-incentive trials

			**Montreal Neurological Institute coordinates**		
**Brain region**	**Voxels**	***Z *** **(maximum)**	***x***	***y***	***z***
Left accumbens	104	6.60	−10	10	−8
Right accumbens/right caudate	57	6.27	8	4	−2
Right amygdala	16	5.65	14	2	−16
Right anterior cingulate gyrus	693	6.73	6	16	34
Right brainstem	386	6.72	8	−28	−18
Left orbital frontal cortex	19	5.74	−34	24	−6
Left insular cortex	82	6.20	−38	14	−8
Right intracalcarine cortex	97	6.47	18	−68	10
Left juxtapositional lobule cortex^a^	55	5.92	−2	0	70
Right lateral occipital cortex^a^	12	6.00	34	−88	−18
Left lingual gyrus	96	6.17	−14	−56	−6
Left occipital pole	67	6.53	−22	−96	16
Right precentral gyrus	22	5.58	44	2	46
Left posterior cingulate	17	5.78	−2	−24	26
Right thalamus	266	7.09	4	−4	2

^a^Two clusters within same region, coordinates and peak activation reported for highest peak activation.

#### rs2268493

**Figure 3:**
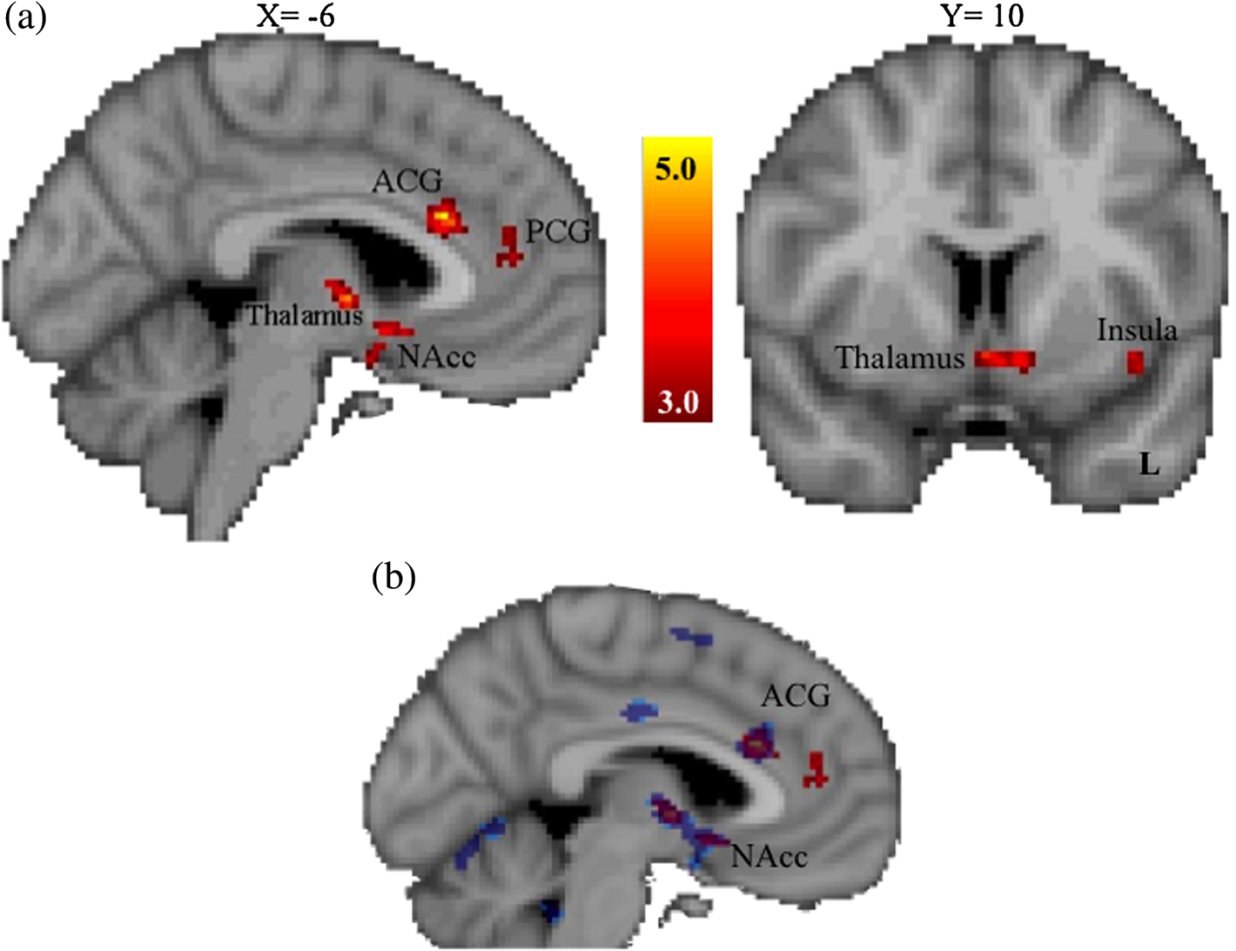
**Brain regions showing significantly reduced activation for the OXTR SNP rs2268493 risk allele group during the anticipation phase of the monetary incentive delay task using both a general linear model approach and permutation testing. (a)** General linear model analyses (in red). *Z* values indicate cluster thresholds. **(b)** Randomize permutation testing analysis (in blue). Note the convergence of effects in the left NAcc and ACG. ACG, anterior cingulate gyrus; L, left; NAcc, nucleus accumbens; OXTR, oxytocin receptor; PCG, paracingulate gyrus; SNP, single nucleotide polymorphism.

During reward anticipation, activation was decreased in risk allele homozygotes (TT) relative to the combined group of heterozygotes and non-risk allele homozygotes (TC/CC) in the left nucleus accumbens, left orbital frontal cortex, right amygdala, bilateral insular cortex, left thalamus and left anterior cingulate gyrus (see Figure 3a and Table 3). Table 3 shows differences in brain activation between allelic groups for the rs2268493 SNP (that is, TT < CC/CT) for incentive versus non-incentive trials during the anticipation phase of the MID task (thresholded at *Z* > 3.0, corrected cluster significance threshold of *P* < .05/3 = 0.0167). No regions showed relatively greater activation for the risk allele homozygotes. These general linear model analyses were then followed up with permutation testing using FSL Randomise to verify allelic differences in key regions of interest. As illustrated in Figure 3b, permutation testing validated the central finding of decreased activity in the left nucleus accumbens and anterior cingulate gyrus.

**Table 3:** Differences in brain activation between allelic groups

			**Montreal Neurological Institute coordinates**		
**Brain region**	**Voxels**	***Z *** **(maximum)**	***x***	***y***	***z***
Left accumbens	83	4.26	−10	10	−10
Right amygdala	65	4.03	12	−4	−18
Left anterior cingulate gyrus^a^	419	4.93	−4	24	24
Brainstem	16	4.24	0	−22	−20
Left orbital frontal cortex^a^	10	3.4	−32	20	−22
Left insular cortex^a^	59	3.87	−32	22	0
Right insular cortex	108	3.66	38	14	−10
Left middle frontal gyrus	21	3.43	−34	10	32
Left occipital fusiform gyrus	147	4.5	−30	−84	−22
Posterior cingulate	11	3.51	0	−44	4
Right superior frontal gyrus^a^	41	3.84	10	2	66
Left thalamus	134	4.58	−6	−4	−2

^a^Two clusters within same region, coordinates and peak activation reported for highest peak activation.

#### rs237887

**Figure 4:**
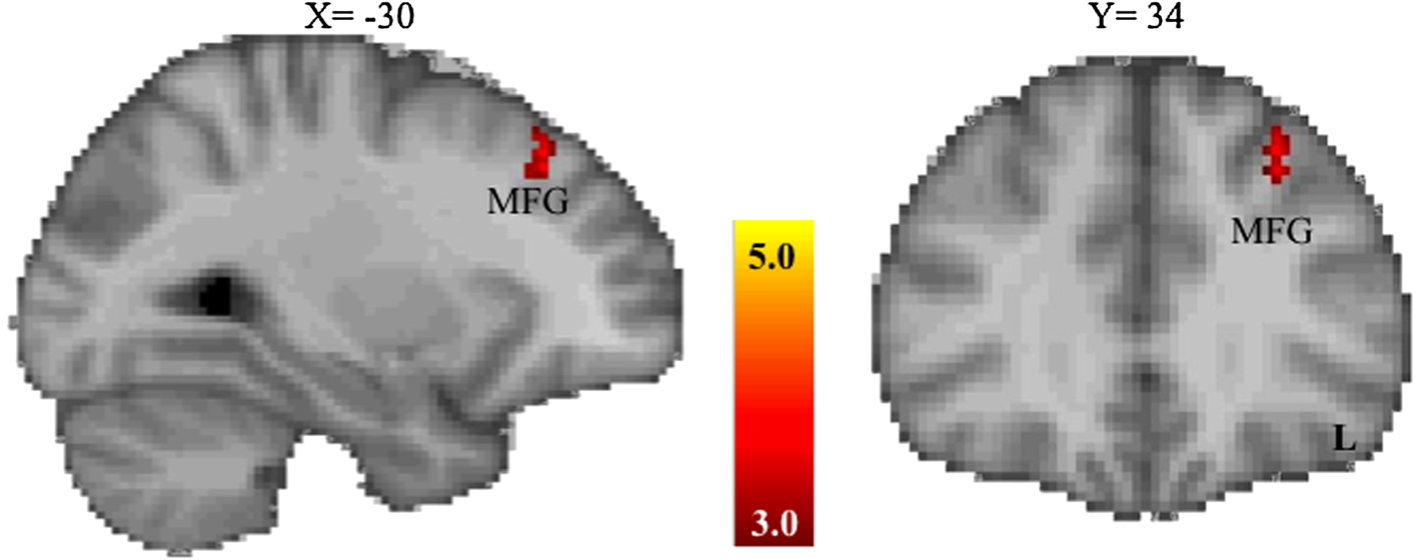
**Brain regions showing significantly reduced activation for the OXTR SNP rs237887 risk allele group the during the anticipation phase of the monetary incentive delay task. ***Z* values indicate cluster thresholds. L, left; MFG, middle frontal gyrus; OXTR, oxytocin receptor; SNP, single nucleotide polymorphism.

Allelic variation of the rs237887 SNP (that is, risk allele homozygotes versus the combined heterozygotes and non-risk homozygotes group) did not significantly influence activation in mesolimbic regions (see Figure 4 and Table 4). Table 4 shows differences in brain activation between allelic groups for the rs237887 SNP (that is, AA < AG/GG) for incentive versus non-incentive trials during the anticipation phase of the MID task (thresholded at *Z* > 3.0, corrected cluster significance threshold of *P* < .05/3 = 0.0167).

**Table 4:** Differences in brain activation between allelic groups

			**Montreal Neurological Institute coordinates**		
**Brain region**	**Voxels**	***Z *** **(maximum)**	***x***	***y***	***z***
Left inferior frontal gyrus (pars opercularis)	27	3.47	−54	14	10
Right lateral occipital cortex	27	3.74	48	−72	−2
Right lateral occipital pole	12	3.36	36	−84	16
Right lingual gyrus	20	3.79	8	−86	−12
Left middle frontal gyrus^a^	39	3.81	−28	32	44
Right occipital fusiform gyrus	68	3.6	14	−78	−14
Right occipital pole	65	4.44	12	−94	16
Right precuneus	36	3.92	6	−56	40
Left supramarginal gyrus (posterior)	60	3.55	−50	−42	46

^a^Two clusters within same region, coordinates and peak activation reported for highest peak activation.

#### rs1042778

Allelic variation of the rs1042778 SNP (that is, risk allele homozygotes versus the combined heterozygotes and non-risk homozygotes group) did not significantly influence activation in mesolimbic regions.

### fMRI data: outcome phase

Allelic variations in the rs2268493, rs237887 and rs1042778 SNPs did not moderate activation of mesolimbic regions during reward outcomes (see Additional file 1 for outcome phase results).

## Discussion

The purpose of this study was to investigate whether allelic variations of OXTR SNPs influence mesolimbic brain activation during reward processing. We found that allelic variations in rs2268493, but not rs237887 or rs1042778, were related to mesolimbic activation during reward anticipation, but not reward outcomes, in healthy adults. The regions moderated by this OXTR SNP, including the nucleus accumbens, amygdala, thalamus, insula, orbital frontal cortex and anterior cingulate gyrus, have been consistently linked to reward motivation. For reviews, see [79,80]. In contrast to brain activation findings, no differences between allelic groups were observed in behavioral responses during the MID task. These findings suggest a disconnect between behavioral and neural responses and that the effects of rs2268493 variations are relatively weaker at the level of behavior than at the level of brain-based endophenotypes [81,82].

The association between rs2268493 and mesolimbic response during reward anticipation is consistent with preclinical evidence that the OXT and mesolimbic dopamine systems act reciprocally and become linked through early social experiences [40,42,43,83-85] and that OXT modulation may directly result in increased firing of dopaminergic neurons in the ventral striatum [41]. Additionally, in humans, exogenous OXT administration influences mesolimbic responses to social stimuli [44,45,86-91] and plasma OXT levels are associated with striatal responses to a social reward (that is, reciprocated cooperation) [45,46].

Understanding the moderating effects of the OXTR gene in the general population may guide the study of genetic mechanisms underlying motivation deficits in ASDs (cf. [92]). This approach of examining the effects of gene variants irrespective of clinical diagnosis can be informative given that risk alleles related to a particular disorder may have a more direct influence on relevant neurobiological endophenotypes than the disorder itself [81]. Thus, gaining an understanding of the effects of risk genes in non-clinical samples may ultimately help to parse the neurobiological heterogeneity of complex neurodevelopmental disorders, such as ASDs [4].

Given the heterogeneity of ASDs and given that hundreds of different genes may be implicated in ASDs with each contributing small degrees of risk [2,92-94], it is important to emphasize that the clinical generalizability of the present findings is contingent on future studies examining the moderating influence of OXT on the mesolimbic response to rewards in individuals with ASDs. In addition, it is important to note that a comprehensive account of candidate molecular genetic influences on brain function in ASDs will require a systems-level approach that accounts for gene–gene interactions, the influence of epistasis and epigenetics on multiple neural networks and the pleiotropic effects of genes on neural phenotypes [95]. Additionally, the present study examined only mesolimbic responses to monetary rewards whereas ASDs have been associated with atypicalities in the processing of both social and non-social rewards [96]. Further research will be necessary to address OXT modulation of mesolimbic responses to social rewards, as suggested by alternative models of oxytocinergic function (for example, [97,98]), and to compare directly the effects of OXTR genes on social and non-social reward motivation in ASDs.

We also note that caution is warranted when interpreting null findings during the outcome phase of the task given the use of a recessive genetic model (that is, comparing risk allele homozygotes with other participants). This model was chosen because it is a more conservative test of the effects of the risk allele on the neural endophenotype and because our sample did not have sufficient numbers of non-risk allele carriers for meaningful analyses that compared risk allele carriers versus non-risk homozygotes (for rs2268493, only two participants were not T-allele carriers).

An important limitation of the present study was the relatively modest sample size, particularly in light of a number of recent non-replications of neuroimaging genetics studies [99]. Thus it may be that modestly sized samples in the context of candidate-gene studies are underpowered [100]. Further, a recent meta-analysis failed to confirm relations between OXTR SNPS rs53576 and rs2254298 and measures of sociability [101]. Accordingly, replication will be necessary to draw firm conclusions about the role of rs2268493 in reward motivation. However, consistent with recommendations for imaging genetics studies [102], we used a well-validated fMRI task that had previously shown robust effects and significant variance in the typical population [103] and participants in the present study were homogeneous in terms of racial and ethnic background, age, IQ and mental health status. Although this sampling strategy limits the generalizability of results, it helps to control for the effects of these non-genetic factors [102]. It is also important to note that OXTR may not be directly related to reward motivation, but rather this relation may be mediated by other functions of the OXT system, such as anxiolytic effects (for example, [38,104]) or effects on memory (for example, [105]). Additionally, the activation clusters associated with allelic differences in rs2268493 were not confined to mesolimbic regions but rather included several regions that are not commonly associated with reward motivation (for example, the brainstem and superior frontal gyrus). Although such findings, which were not hypothesized, indicate that the moderating effects of rs2268493 may not be constrained to mesolimbic regions, future studies will be needed to replicate these results.

## Conclusions

Despite these limitations and interpretive cautions, this study is a preliminary step towards a better understanding of the potential genetic mechanism underlying individual differences in neural activation during reward motivation, which may ultimately contribute to our understanding of dysfunctional motivational systems in ASDs. Although identifying brain functions associated with specific genes is an important early step in understanding the effects of particular ASD risk genes on ASD symptom expression, the link between a single gene and brain function will ultimately only explain a small part of the complex genetic heterogeneity of ASDs [97]. A systems approach to studying genetic and brain imaging data will be necessary for integrating information about the effects of multiple alleles on multiple genes, multigenic interactions on neural networks, pleiotropic effects of genes on cognitive, affective, and motor development, and the influence of environmental and epigenetic factors on these processes.

## Abbreviations

AQ: autism spectrum quotient; ASD: autism spectrum disorder; BOLD: blood oxygen level dependent; BRAVO: brain volume imaging; DSM-V: Diagnostic and Statistical Manual of Mental Disorders (Fifth Edition); FEAT: FMRI expert analysis tool; FLAME: FMRIB's Local Analysis of Mixed Effects; FLIRT: FMRIB Linear Image Registration Tool; fMRI: functional magnetic resonance imaging; FMRIB: functional magnetic resonance imaging of the brain; FSL: FMRIB software library; IQ: intelligence quotient; MCFLIRT: motion correction and FMRIB's linear image registration tool; MID: monetary incentive delay; NART-R: National Adult Reading Test – Revised; OXT: oxytocin; OXTR: oxytocin receptor; SCID- I: Structured Clinical Interview for DSM-IV Axis I Disorders; SCL-90-R: Symptom Checklist-90-Revised; SENSE: sensitivity encoding; SNP: single nucleotide polymorphism; SPGR: spoiled gradient echo.

## Competing interests

The authors declare that they have no competing interests.

## Authors’ contributions

CRD coordinated the study, screened participants, collected data, analyzed behavioral and fMRI data, and drafted the manuscript. JA was involved in the collection and analysis of data and helped to draft the manuscript. KD, CJB, RVK and REM were involved in participant recruitment, collection of data and revising the manuscript. MGM, AAK, OAM, RMC and SAH were involved in the interpretation of data and drafting the manuscript. FJM helped to design and coordinate the study, interpret the data and draft the manuscript. GSD conceived, designed and coordinated the study, and helped to analyze the data and draft the manuscript. All authors read and approved the final manuscript.

## Supplementary Material

Additional file 1 **fMRI results from the outcome phase of the monetary incentive delay task.** This document details the results of analyses involving brain activation during the outcome phase of the monetary incentive delay task for the entire sample and each of the three SNPs (rs2268493, rs1042778 and rs237887). Tables displaying the regions with significant activation during the outcome phase for the entire sample and for the SNP rs237887 are also included. Click here for file
