# Supplementary material for: Association between the oxytocin receptor (OXTR) gene and mesolimbic responses to rewards
Source: Mol Autism. 2014 Jan 31;5:7. doi: 10.1186/2040-2392-5-7 (PMC3922109; doi:10.1186/2040-2392-5-7)
Supplement: Additional file 1 — fMRI results from the outcome phase of the monetary incentive delay task. This document details the results of analyses involving brain activation during the outcome phase of the monetary incentive delay task for the entire sample and each of the three SNPs (rs2268493, rs1042778 and rs237887). Tables displaying the regions with significant activation during the outcome phase for the entire sample and for the SNP rs237887 are also included. [file 2040-2392-5-7-S1.docx]

Additional file for “Association between the Oxytocin Receptor (OXTR) Gene and the Mesolimbic Response to Rewards,” by Damiano, Aloi, Dunlap, Burrus, Kozink, McLaurin. Mullette-Gillman, Carter, Huettel, McClernon, & Dichter

**fMRI Results from the Outcome Phase of the Monetary Incentive Delay Task**

**fMRI Data: Outcome Phase**

**Entire Sample.** Across the entire sample, regardless of allelic group, no regions showed significant activation at a threshold of Z> 5.25 (the threshold used for reporting the anticipation phase results). However at Z> 3.0, activation was observed in the paracingulate/frontal medial cortex and anterior cingulate gyrus (see Table 1).

**rs2268493.** No regions showed activation differences between risk allele homozygotes (TT) and the combined heterozygotes and non-risk homozygotes (TC/CC) group during reward outcomes.

**rs237887.** No mesolimbic reward processing regions showed increased or decreased activation in the risk allele homozygotes (AA) relative to the combined heterozygotes and non-risk allele homozygotes (AG/GG) group during reward outcomes (see Table 2).

**rs1042778.** No regions showed activation differences between risk allele homozygotes (GG) and the combined heterozygotes and non-risk allele homozygotes (TT/TG) group during reward outcomes.

Table S1: *Activation during reward versus non-reward outcome trials across the entire sample (thresholded at Z > 3.0, corrected cluster significance threshold of p < .05).*

|  |  |  | **MNI Coordinates** | | |
| --- | --- | --- | --- | --- | --- |
| **Brain Region** | **Voxels** | **Z (Max)** | **X** | **Y** | **Z** |
| R Anterior Cingulate Gyrus | 330 | 4.51 | 4 | 36 | 14 |
| R Brain-Stem^a^ | 33 | 4.44 | 4 | -28 | -56 |
| R Intracalcarine Cortex^a^ | 38 | 3.92 | 20 | -64 | 8 |
| L Lingual Gyrus | 15 | 4.33 | -8 | -42 | -8 |
| R Paracingulate Gyrus/ Frontal Medial  Cortex | 15 | 3.54 | 4 | 44 | -8 |
| R Posterior Cingulate | 52 | 3.85 | 4 | -16 | 32 |

*Note.* All coordinates (x, y, z) are given in MNI space. L: left, R: right.

^a^Two clusters within same region, coordinates and peak activation reported for highest peak activation.

Table S2: *Differences in brain activation between allelic groups for the rs237887 SNP (i.e., AA<AG/GG) for reward versus non-reward outcome trials (thresholded at Z > 3.0, corrected cluster significance threshold of p < .05/3=0.166).*

|  |  |  | **MNI Coordinates** | | |
| --- | --- | --- | --- | --- | --- |
| **Brain Region** | **Voxels** | **Z (Max)** | **X** | **Y** | **Z** |
| L Intracalcarine Cortex | 92 | 4.11 | -10 | -76 | 8 |
| R Lateral Occipital Cortex^a^ | 42 | 3.69 | 46 | -80 | -2 |
| R Lingual Gyrus | 20 | 3.87 | 8 | -86 | -14 |
| L Occipital Fusiform Gyrus | 31 | 4.04 | -24 | -68 | -14 |
| R Occipital Fusiform Gyrus^a^ | 46 | 4.13 | 46 | -64 | -28 |
| L Occipital Pole | 22 | 3.14 | -30 | -92 | 10 |

*Note.* All coordinates (x, y, z) are given in MNI space. L: left, R: right.

^a^Two clusters within same region, coordinates and peak activation reported for highest peak activation.
